# Enhanced Expression of Integrin αvβ3 Induced by TGF-β Is Required for the Enhancing Effect of Fibroblast Growth Factor 1 (FGF1) in TGF-β-Induced Epithelial-Mesenchymal Transition (EMT) in Mammary Epithelial Cells

**DOI:** 10.1371/journal.pone.0137486

**Published:** 2015-09-03

**Authors:** Seiji Mori, Moe Kodaira, Ayano Ito, Mika Okazaki, Naomasa Kawaguchi, Yoshinosuke Hamada, Yoshikazu Takada, Nariaki Matsuura

**Affiliations:** 1 Department of Molecular Pathology, Osaka University Graduate School of Medicine, Division of Health Sciences, 1–7 Yamada-oka, Suita-shi, Osaka, 565–0871, Japan; 2 Departments of Dermatology, Biochemistry and Molecular Medicine, School of Medicine, University of California Davis, Sacramento, California, 95817, United States of America; 3 Graduate Institute of Translational Medicine, College of Medical Science and Technology, Taipei Medical University, 520 Wu-Hsing Street, Taipei, 11031, Taiwan, R.O.C; Florida International University, UNITED STATES

## Abstract

Epithelial-to-mesenchymal transition (EMT) plays a critical role in cancer metastasis, and is regulated by growth factors such as transforming growth factor β (TGF-β) and fibroblast growth factors (FGF) secreted from the stromal and tumor cells. However, the role of growth factors in EMT has not been fully established. Several integrins are upregulated by TGF-β1 during EMT. Integrins are involved in growth factor signaling through integrin-growth factor receptor crosstalk. We previously reported that FGF1 directly binds to integrin αvβ3 and the interaction was required for FGF1 functions such as cell proliferation and migration. We studied the role of αvβ3 induced by TGF-β on TGF-β-induced EMT. Here, we describe that FGF1 augmented EMT induced by TGF-β1 in MCF10A and MCF12A mammary epithelial cells. TGF-β1 markedly amplified integrin αvβ3 and FGFR1 (but not FGFR2). We studied if the enhancing effect of FGF1 on TGF-β1-induced EMT requires enhanced levels of both integrin αvβ3 expression and FGFR1. Knockdown of β3 suppressed the enhancement by FGF1 of TGF-β1-induced EMT in MCF10A cells. Antagonists to FGFR suppressed the enhancing effect of FGF1 on EMT. Integrin-binding defective FGF1 mutant did not augment TGF-β1-induced EMT in MCF10A cells. These findings suggest that enhanced integrin αvβ3 expression in addition to enhanced FGFR1 expression is critical for FGF1 to augment TGF-β1-induced EMT in mammary epithelial cells.

## Introduction

Dynamic cross-regulation of growth factors is a hallmark of epithelial-mesenchymal transition (EMT) [1–3]. Fibroblast growth factors (FGFs) control multiple biological processes such as proliferation, survival, migration and differentiation of a variety of cell types [4, 5]. FGF signaling also plays a role in EMT and morphogenesis of mesoderm in mice at gastrulation by controlling Snail that inhibits E-cadherin expression [6]. Thus FGF signaling is necessary to control a specification of mesodermal and endodermal fates through some of the genes involved in the EMT during development [7].

Deregulation of FGF signaling in different types of cancer has been reported. In most cases FGF signaling is overactivated by constitutively active mutations of FGF receptors, gene amplifications, and autocrine and paracrine signaling [8]. Aberrant FGF signaling promotes tumor development by directly regulating cancer cell proliferation, survival, and by promoting tumor angiogenesis [9].

FGF1 is a prototypic member of the FGF family, which has been implicated in a range of physiological processes, including development; wound healing and cancer development [10]. Ectopic expression of FGF1 in bladder carcinoma cells induces a mesenchymal phenotype correlated with the internalization of E-cadherin and the relocation of β-catenin from the cell membrane to the cytoplasm and nucleus [11]. Stimulation of the bladder cells with FGF1 induces a set of genes attributed to EMT induction and to proteolysis [12]. FGF1 reverts TGF-β1-induced EMT in human and rat alveolar epithelial-like cell lines [13]. The role of FGF1 and the molecular mechanisms by which FGF1-regulated EMT during cancer progression remain unsolved.

Integrins are a family of cell adhesion receptors that recognize extracellular matrix ligands, cell surface molecules, and growth factors. Integrins are transmembrane α-β heterodimers, and at least 18 α and 8 β subunits are known [14]. In addition to mediating cell adhesion, integrins make transmembrane connections to the cytoskeleton and activate several intracellular signaling pathways [15]. Integrin signaling and functions are dependent on crosstalk with other signaling pathways, especially growth factor signaling pathways, since integrins possess no enzymatic activity [16, 17]. Several integrins are upregulated by TGF-β1 during the EMT process [18, 19]. It has been well established that integrins are involved in growth factor signaling through integrin-growth factor receptor crosstalk. We have previously demonstrated that FGF1 directly interacts with integrin αvβ3 and induces the FGF1-FGF receptor (FGFR)-integrin αvβ3 ternary complex formation [20–22]. This interaction is necessary for FGF1 functions including cell proliferation, migration and angiogenesis [20–22].

TGF-β1 induces integrin αvβ3 expression and the enhanced expression of αvβ3 potentiates TGF-β1-induced responses in lung fibroblasts [23, 24]. Also, integrin αvβ3 enhances TGF-β pathway through TβR-II activation and enhances EMT in mammary epithelial cells [25]. Furthermore, integrin αvβ3 induces metastatic phenotype in hepatocellular carcinoma by enhancing TGF-β1 signaling [26]. However, the precise role of αvβ3 in TGF-β1-induced EMT has not been established.

In this study we studied the effect of FGF on TGF-β-induced EMT in mammary epithelial cells. In this model TGF-β induces αvβ3 and FGF1 enhanced TGF-β-EMT. We demonstrate that direct binding of FGF1 to αvβ3 is required for the enhancing effect of FGF1 on TGF-β-induced EMT. So the enhanced expression of αvβ3 is a critical component in the enhancing effect of FGF1 on TGF-β-induced EMT. This represents a new model of growth factor-integrin crosstalk in EMT.

## Materials and Methods

### Cell Culture and Treatments

MCF10A and MCF12A human mammary epithelial cell line, SK-BR-3 and ZR-75-30 breast cancer cell lines were obtained from American Type Culture Collection (ATCC). MCF10A and MCF12A cells were cultured in DMEM/F-12 containing 5% horse serum, 100 U/ml penicillin, 100 μg/ml streptomycin, and 10 μg/ml insulin, 20 ng/ml EGF, 1 ng/ml cholera toxin, 100 μg/ml hydrocortisone [27]. SK-BR-3 cells stably knockdown integrin β3 and ZR-75-30 cells that stably express integrin β3 were cultured in DMEM and RPMI-1640, respectively, and supplemented with 10% fetal bovine serum, 100 U/ml penicillin and 100 μg/ml streptomycin. Experiments were performed when cells reached 50 to 60% confluence. Recombinant human TGF-β1 was purchased from PeproTech (Rocky Hill, NJ) and reconstituted in water according to the manufacturer's instructions. Wild-type FGF1 (WT) and mutant form FGF1 (R50E) were bacterially expressed and purified as described previously [20]. FGFR inhibitor PD173074 and MEK inhibitor U0126 were purchased from Wako pure chemicals (Osaka, Japan) and dissolved in Me_2_SO (DMSO). To induce EMT, cells were starved for 8 to 16 h then stimulated with 5 ng/ml TGF-β1 in serum-free medium and/or 50 ng/ml recombinant human FGF1 (WT or R50E) plus heparin (10 μg/ml) and additionally incubated for 48hous.

### Transient and Stable Transfection of Integrin β3

For transient gene silencing experiments, Mission siRNA oligonucleotides (Sigma) for the human integrin β3 (catalog no. SASI_Hs01_00174219 and SASI_Hs01_00174221) were used. Mission siRNA universal negative control (Sigma) was used as a negative control. The siRNAs, 30 nM final concentration of each siRNA duplex, were transfected into MCF10A cells using the lipofectoamine RNAiMAX (Invitrogen) according to the manufacturer's instructions. After a 48 h gene silencing for Integrin αv and β3, respectively, both target gene and control siRNA-treated cells were harvested and applied for Western blotting or flow cytometry.

Stably transfectable anti-integrin β3 short hairpin RNA plasmid was generated as follows. We synthesized oligodeoxyribonucleotide chosen from The RNAi Consortium (TRC) shRNA library (Broad Institute) for integrin β3 (Clone ID. TRCN0000003236). The shRNA segment was subcloned downstream of the human U6 promoter between restriction sites AgeI and EcoRI in the pLKO.1-TRC cloning vector (Addgene), which contains puromycin resistance marker. The scramble shRNA (Addgene) were used as negative control.

For stable transfection of integrin β3, pcDNA3.1-β3 (Addgene) that contains neomycin resistance marker was used. Empty pcDNA3.1 vector was used as a negative control. The plasmids were transfected into SK-BR-3 cells for knock-down or ZR-75-30 cells for overexpressing using lipofectamine 2000 (Invitrogen) according to the manufacturer's instructions and selected successful integration by puromycin or G418 for 10 days. For generation of clonal stable cell lines, single colonies were chosen and propagated in the presence of puromycin or G418 containing media, and expression of integrin β3 was confirmed by Western blotting and dimerization of integrin αv and β3 in ZR-75-30 cells was confirmed by flow cytometry using antibody LM609 that is specific to αvβ3 heterodimer.

### Three-Dimensional Culture Assays-MCF10A Cells Form Acini when Cultured on Matrigel In Vitro

The constitute cells can establish apicobasal polarity and form a hollow sphere [28, 29]. MCF10A cells were seeded on glass cover slip coated with growth factor–reduced Matrigel (BD Biosciences), as described [27]. Cells were cultured in assay medium (DMEM/F12 supplemented with 2% horse serum, 10 μg/mL insulin, 1 ng/mL cholera toxin, 100 μg/mL hydrocortisone, 50 units/mL penicillin and 50 μg/mL streptomycin) containing 5 ng/mL EGF and 2% growth factor-reduced Matrigel. Medium was changed every 4 days. After 2 weeks of incubation, acinar structures were formed on the Matrigel. Assay medium was replaces to DMEM/F12 containing 2% horse serum and 2% growth factor-reduced Matrigel. Then EMT was induced by 5 ng/ml TGF-β1 and 50 ng/ml recombinant human FGF1 (WT or R50E) for 4 days. The medium was changed once during period of stimulation.

### Western Blotting

Cells were lysed in lysis buffer (10 mM Tris-HCl pH 7.5, 150 mM NaCl, 1 mM EDTA, 0.5% NP-40, protease inhibitor cocktail and phosphatase inhibitor cocktail (Nacalai, Kyoto, Japan)). Samples were resolved by SDS-PAGE and transferred to a polyvinylidene difluoride membrane (Millipore, Billerica, MA). The blots were proved with primary antibodies and horseradish peroxidase conjugated secondary antibodies (GE healthcare, Piscataway, NJ), and then visualized by enhanced chemiluminescence reagent (Pierce, Rockford, IL). Antibodies used for Western blotting were as follows. Rabbit anti-N-cadherin (YS) was Immuno-Biological Laboratories (Gunma, Japan); mouse anti-E-cadherin (4A2C7) was Invitrogen (Carlsbad, CA); rabbit anti-ERK1/2 (137F5), rabbit anti-phospho ERK1/2 (D13.14.4E), rabbit anti-phospho Smad2, mouse anti-MMP9 (D603H), mouse anti-PAI-1 (D9C4) were from Cell Signaling Technology (Danvers, MA); rabbit anti-FGFR1 (C-15), mouse anti-integrin β3 (SAP), mouse-integrin αV (P2W7), mouse anti-GAPDH (0411) were from Santa Cruz Biotechnology (Santa Cruz, CA); rabbit anti- FGFR2 was from Sigma; mouse anti-MMP2 (42-5D11) was from Daiichi fine chemical (Toyama, Japan). Densitometric analysis of Western blots was performed using ImageJ software (http://rsb.info.nih.gov/ij/index.html).

### Reverse Transcription Quantitative Real-Time PCR

Total RNA was isolated using Sepasol (Nacalai, Kyoto, Japan), cDNA was synthesized using AMV Reverse Transcriptase (Promega, Madison, WI) and subsequent quantitative real-time PCR was done in a Roche LightCycler-480 using SYBR Green Master Mix (Roche, Indianapolis, IN) according to manufacturer’s instructions. Expressions were quantified using the following primers: N-cadherin 5’-CCATCACTCGGCTTAATGGT-3’ and 5’-ACCCACAATCCTGTCCACAT-3’ and vimentin 5’-GCCCTTAAAGGAACCAATG-3’ and 5’-CCTTCCAGCAGCTTCCTGTAG-3’, Snail1 5’- GGTTCTTCTGCGCTACTGCT-3’ and 5’- TAGGGCTGCTGGAAGGTAAA-3’ and, Snail2 5’- GCGATGCCCAGTCTAGAAAA-3’ and 5’- GCAGTGAGGGCAAGAAAAAG-3’. Relative quantification of cDNA amounts was done using the data analysis function of the Roche Molecular Biochemicals Light Cycler Software (V. 3.5). Expression levels were calculated in relation to the expression level of β-Actin and glyceraldehyde-3-phosphate dehydrogenase (GAPDH) as the reference genes. The primers for β-Actin were 5’-CTCCTCCCTGGAGAAGAGCTACGA- 3’ and 5’-ATGATGGAGTTGAAGGTAGTTTCG- 3’ and GAPDH were 5’-CAATGACCCCTTCATTGACC-3’ and 5’-TTGATTTTGGAGGGATCTCG-3’. For quantification using real-time reverse transcription-PCR, at least three independent experiments with triplicate amplifications were performed.

### Immunofluorescence

Cells were seeded and treated on coverslips. They were then fixed in 4% paraformaldehyde for 10 minutes, permeabilized with 0.5% Triton X-100 in PBS for 10 minutes, washed twice with PBS, and blocked with 1% BSA in PBS for 1 hour, cells were incubated with primary antibodies diluted in blocking buffer. Primary antibodies used for immunofluorescence as follows. Mouse anti-integrin αvβ3 (LM609, Millipore), mouse anti-laminin-5 (D4B5, Millipore) and rat anti-integrin α6 (GoH3, eBioscience). Secondary antibodies were donkey anti-mouse IgG FITC conjugated (GE healthcare) and donkey anti-rat IgG FITC conjugated (GE healthcare). Coverslips were mounted in Prolong Gold with 4′,6-diamidino-2-phenylindole (DAPI; Invitrogen). Fluorescence images were taken by Nikon Eclipse TE2000E fluorescence microscope (Nikon, Tokyo, Japan) with digital camera.

### Flow Cytometry

For flow cytometric detection of integrins, treated cells grown in culture dishes were harvested with Trypsin/EDTA and washed with PBS. After incubation in a primary antibody in 1% BSA in DMEM for 30 minutes at 4°C, the cells were washed with 1% BSA in DMEM. Primary antibodies used in this assay were anti-integrin αvβ3 (LM609), anti-integrin β1 (SG19), anti-integrin β3 (SAP), anti-integrin α2 (P1E6, Millipore), anti-integrin α3 (P1B5, Santa Cruz Biotechnology), anti-integrin α4 (SG73), anti-integrin α5 (KH72), anti-integrin α6 (GoH3) and anti-integrin α9β1 (Y9A2, Santa Cruz Biotechnology). Then cells were incubated in FITC conjugated secondary antibody in 1% BSA in DMEM. Fluorescence-activated cell sorting (FACS) analysis (FACS Calibur, Becton Dickinson, San Jose, CA) was done after additional washes in PBS at 4°C.

### Invasion Assay

Invasion assays were done in Chemotaxicell inserts (Kurabo, Osaka, Japan) with 8 μm polycarbonate filters coated with 100 μg/ml Growth factor reduced Matrigel for 3 h at 37°C and blocked with 0.1% bovine serum albumin in PBS. Cells were suspended in serum-free DMEM/F12 containing 0.1% BSA at a concentration of 10^5^ cells in 200 μl and seeded in the upper chamber. The lower chamber was filled with 500 μl DMEM/F12 containing 0.1% BSA and 5 ng/ml TGF-β1 and/or 50 ng/ml recombinant human FGF1. Then, cells were allowed to migrate for 24 h. The upper side of the filters was wiped with cotton swabs, fixed, and stained with 0.1% crystal violet. Images were taken by digital camera and counted using cell counting function of Image J software.

### Gelatin Zymography

Conditioned media were collected and concentrated using a Centricon (Millipore, Bedford, MA). Gelatinolytic activities were detected in SDS-polyacrylamide gels containing 2 mg/ml gelatin. After electrophoresis, SDS was removed by incubating the gel with 2.5% Triton X-100 and gelatinase activity was revealed by overnight incubation at 37°C with incubation buffer containing 50 mM Tris-HCl pH 6.8, 5 mM CaCl_2_, 1 μM ZnCl_2_. Then the gel was stained with Coomassie Brilliant Blue.

### In Vitro Wound Healing Assay

Cells were allowed to grow in the culture medium for overnight in cell culture dishes. Then cells were washed with serum-free medium and starved for 8 h, and treated with 5 ng/ml TGF-β1 and/or 50 ng/ml recombinant human FGF1 (WT or R50E) to induce EMT for 24 h. A scratch was made across the cell layer using a pipette tip. After washing with serum-free medium twice, cells were incubated in same conditioned as above for additional 24 h. Images were observed using phase-contrast inverted microscope (Olympus, Tokyo, Japan) with digital camera and the wounded areas were quantified by using ImageJ.

### Statistical Analysis

Results are presented as the mean ± S.E. of at least three independent experiments. Significant differences were defined as *p* < 0.05.

## Results

### FGF1 Enhances TGF-β1-Induced EMT in Mammary Epithelial Cells

**Fig 1:**
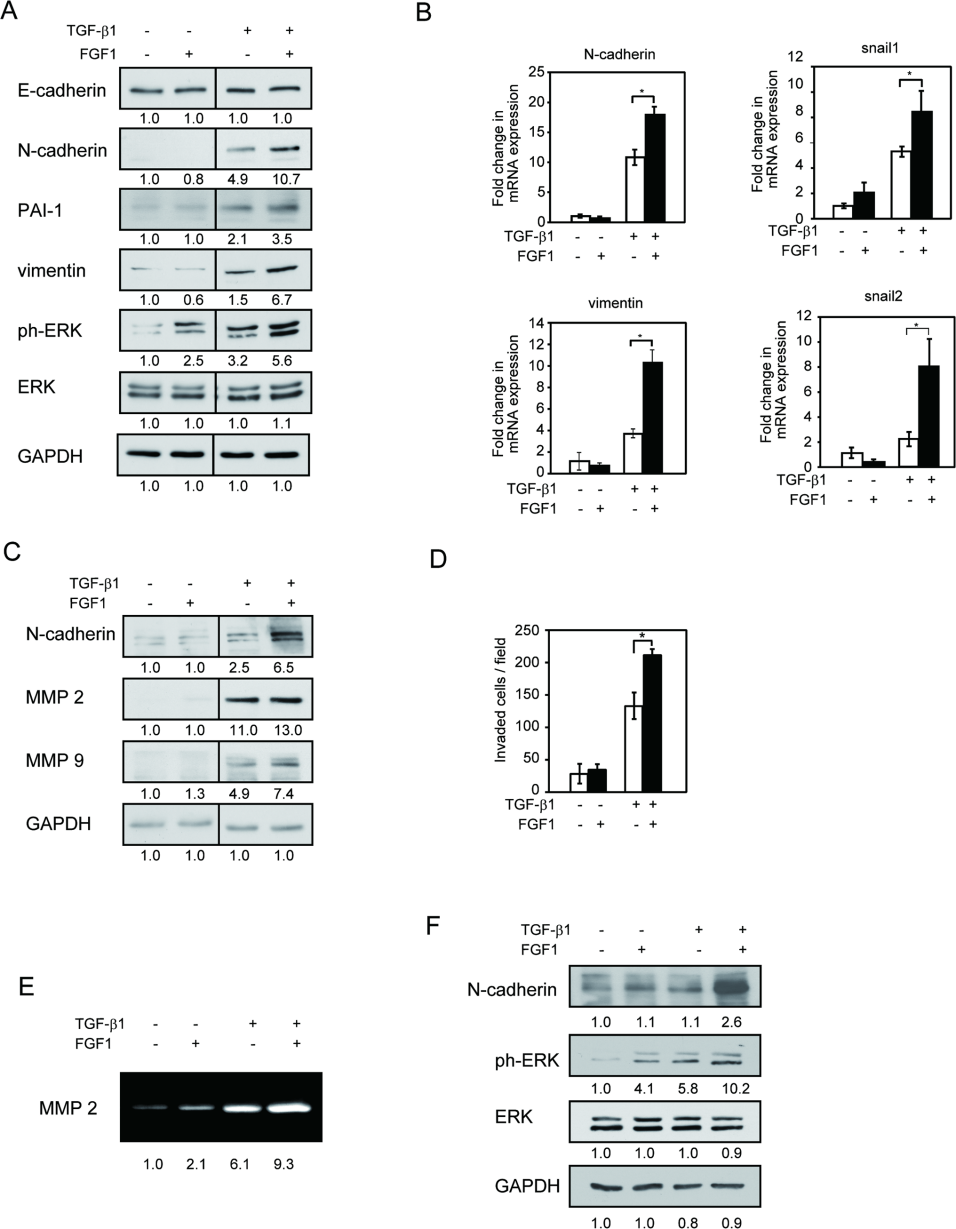
FGF1 amplifies TGFβ-1-induced EMT in mammary epithelial cells. Starved MCF10A cells were treated with or without 5 ng/ml TGF-β1 in the absence or presence of 50 ng/ml FGF1 for 48 h. *A*, Cell lysates were applied for Western blotting with the indicated antibodies. GAPDH and total ERK1/2 were used as a loading control. *B*, Total RNA was extracted and revers transcribed to cDNA. N-cadherin and vimentin, Snail1 and Snail2 expressions were assessed by real-time RT-PCR. *C*, Cell lysates were applied for Western blotting with the indicated antibodies. *D*, Matrigel invasion assay was performed on MCF10A cells following treatment with 5 ng/ml TGF-β1 in the presence or absence of FGF1. *E*, Conditioned media were applied for gelatin zymography and MMP2 activity were assessed by gelatin digestion in the gel. *F*, MCF12A cells were starved and treated with or without 5 ng/ml TGF-β1 in the absence or presence of 50 ng/ml FGF1 for 48 h. Western blotting was performed with the indicated antibodies. Data represent the mean ± S.E. (n = 3; *, *p* < 0.05). Bands intensity was measured by densitometry.

We studied the effect of FGF1 on TGF-β1-induced EMT in MCF10A human mammary epithelial cells. Serum-starved MCF10A cells were incubated for 48 h in serum-free medium containing TGF-β1 with or without 50 ng/ml FGF1. EMT markers, N-cadherin, PAI-1 and vimentin levels were upregulated by TGF-β1 in MCF10A cells. Interestingly, combined stimulation of TGF-β1 plus FGF1 enhanced the protein and mRNA levels of these proteins (Fig 1A and 1B).

Despite E-cadherin is one of the markers of EMT, we did not detect the obvious E-cadherin downregulation by TGF-β1 in this condition (Fig 1A). This is not surprising since 1) not all cell lines that have undergone EMT concomitantly lose E-cadherin expression [30], and 2) the E-cadherin expression is not sufficiently suppressed by TGF-β1 within 48 h TGF-β1 stimulation [31, 32].

It has been reported that TGF receptor and other tyrosine kinase receptors activate extracellular signal-regulated kinase (ERK) [33, 34]. TGF-β1 activated-ERK phosphorylation was also enhanced by FGF1 (Fig 1A). When FGF1 was added after 24 h TGF-β stimulation, we still observed synergistic effects on N-cadherin level and ERK phosphorylation (data not shown). This suggests that the effect of FGF1 on TGF-β-induced EMT is not specific to 48 h incubation time.

We tested the effect of FGF1 on MMP2 and MMP9, target genes of TGF-β1. MMP9 was amplified by combined stimulation of TGF-β1 and 50 ng/ml FGF1, while no significant change was detected in MMP2 expression (Fig 1C).

EMT is also characterized by increased cell motility and invasiveness, key events in cancer metastasis [35]. We examined whether FGF1 regulates TGF-β1 induced cell invasion. MCF10A cells were cultured on Matrigel coated trans-well chamber for 24 h and invaded cells were counted. FGF1 enhanced the invasion of MCF10A cells into Matrigel when cells were treated with TGF-β1 (Fig 1D). Furthermore, consistent with the result of the invasion assay, TGF-β -induced MMP2 secretion was upregulated by FGF1 in gelatin zymography (Fig 1E). We used another normal mammary epithelial cell line, MCF12A, was used to study if the effect of FGF1 on TGF-β1-induced EMT is cell-specific. We obtained very similar results: N-cadherin and phosphorylated ERK were upregulated by combination of FGF1 plus TGF-β1 (Fig 1F), suggesting that the effect of FGF1 on TGF-β1-induced EMT is not cell-specific. To rule out the effect of FGF1 is due to its effect on cell proliferation, we counted the cell number after 48 h stimulation with FGF, TGF-β, or FGF plus TGF-β. There was no difference among the cell numbers (data not shown), suggesting that the observed affects were not due to FGF1’s effect on cell proliferation.

### Integrin αvβ3 Is Markedly Upregulated by TGF-β1

We have previously reported that FGF1, integrin αvβ3 and FGFR1 ternary complex formation is critical for FGF signaling such as motility, cell proliferation, and angiogenesis [20, 21]. Integrin αvβ3 is induced by TGF-β1 in lung fibroblast cells and murine mammary gland cells [23, 25]. Hence, we reasoned that the enhancement effect of FGF1 is due to increased integrin αvβ3 levels.

**Fig 2:**
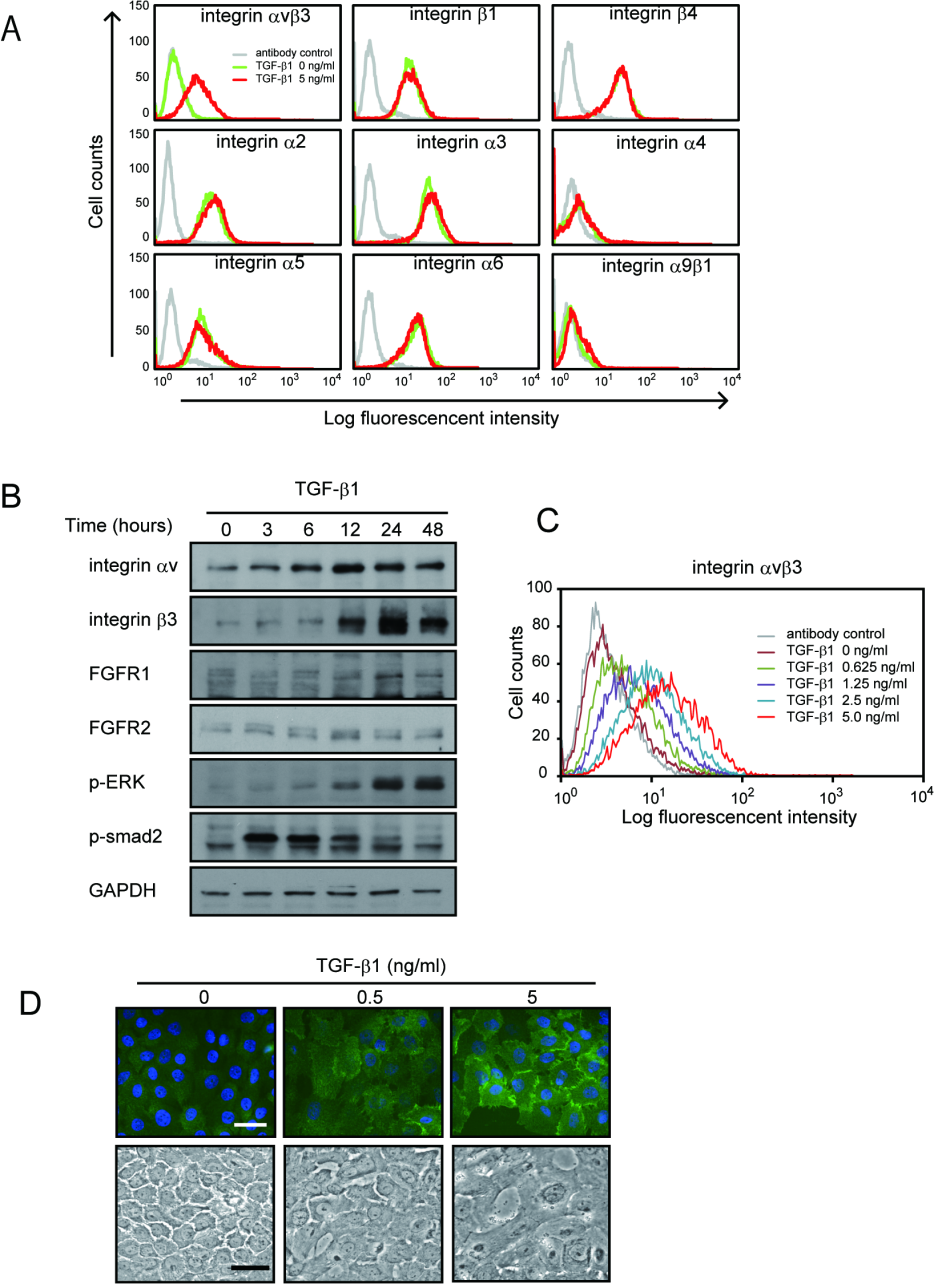
Integrin αvβ3 and FGFR1 are induced by TGF-β1 in MCF10A cells. *A*, Starved MCF10A cells were stimulated with or without 5 ng/ml TGF-β1 for 48 h. Cell surface expression of integrins were determined by FACS analysis using indicated antibodies. *B*, We performed time-course analysis of FGFR1, FGFR2, and integrin αv and β3 expression by Western blotting. Starved MCF10A cells were stimulated with DMSO or TGF-β1 (5 ng/ml) for the indicated time periods. Cell lysates were applied for Western blotting and proved with antibodies shown in figure. GAPDH was used as loading control. *C*, Dose-dependent effect of TGF-β1 on integrin αvβ3 was assessed by FACS flow cytometry. MCF10A cells were treated with the indicated concentration of TGF-β1. *D*, Induction of integrin αvβ3 was determined by immunofluorescence microscopy. Cells were stimulated with TGF-β1 (0.5 or 5 ng/ml) for 48 h, and stained with anti-integrin αvβ3 antibody (LM609) with FITC conjugated secondary antibody to visualize (upper). Lower panels indicate representative phase contrast images of TGF-β1 stimulated cells. Scale bar = 20 μm.

To address this question, we first studied which integrins are induced by TGF-β1 in MCF10A cells. FACS analysis revealed that integrin αvβ3 was the only integrin induced by TGF-β1 among tested integrins (Fig 2A). We next determined the levels of integrin αvβ3 expressions by Western blotting. TGF-β1 quickly and strongly induced integrin αvβ3 (Fig 2B). We also studied the time-course of TGF- β signaling. Interestingly, 5 ng/ml TGF-β1 induced both canonical Smad pathway and non-canonical MAP kinase pathway. Smad2 phosphorylation peaked at 3 h and gradually decreased until 48 h, while phosphorylated ERK peaked at 48 h after TGF-β1 stimulation (Fig 2B). Integrin αvβ3 expression on the cell surface was assessed by flow cytometer using anti-integrin αvβ3 antibody (LM609). Integrin αvβ3 was induced by TGF-β1 dose-dependently in MCF10A cells (Fig 2C). Similar results were obtained by immuno-staining analysis. Integrin αvβ3 was localized to plasma membrane, and the level of integrin αvβ3 was much increased by TGF-β1 (Fig 2D). To clarify the morphological changes of the EMT by the TGF, we included phase contrast images in Fig 2D lower panel. These results suggest that TGF-β1 markedly induces αvβ3 in MCF10A cells.

### Integrin αvβ3 Is Required for FGF1 to Enhance TGF-β1-Induced EMT

**Fig 3:**
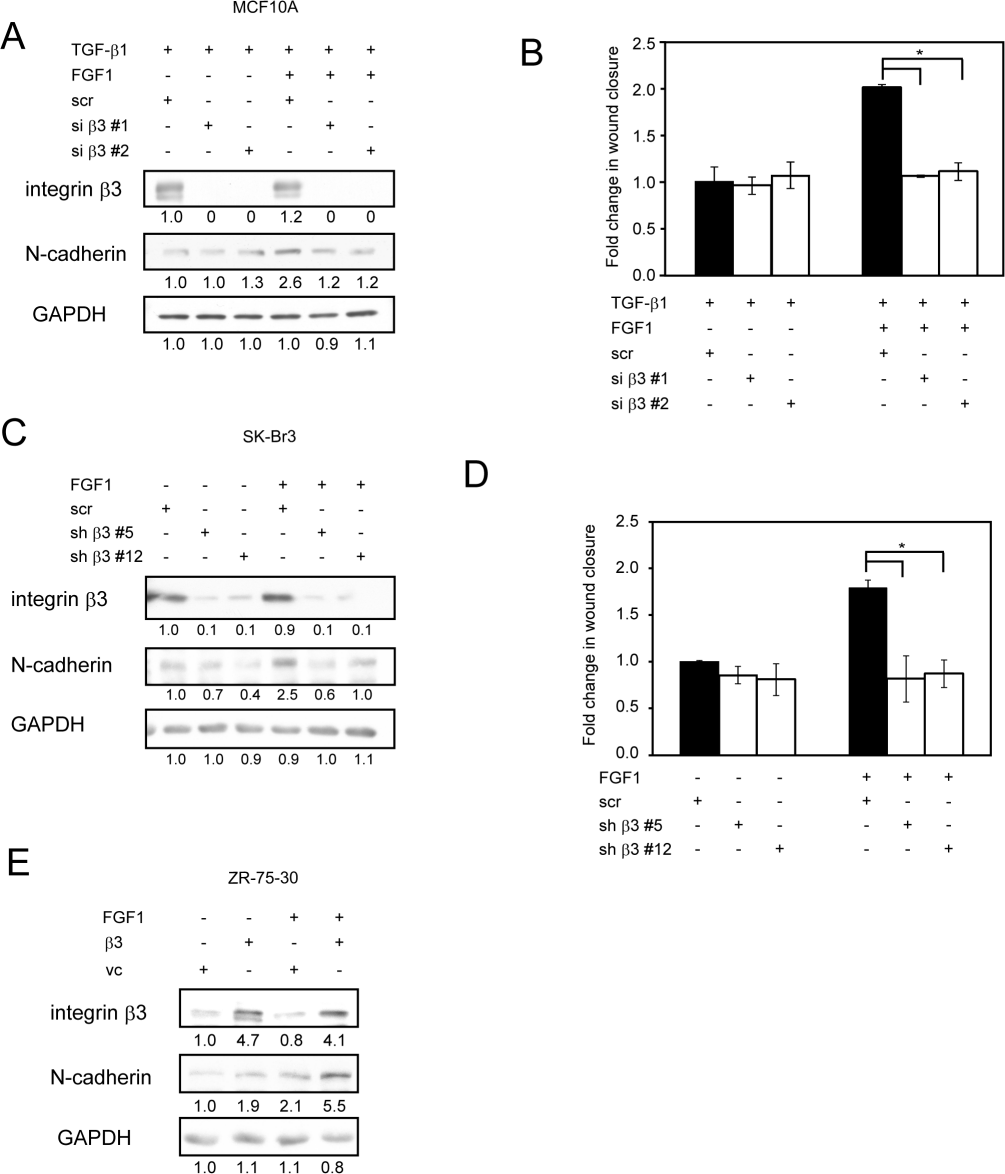
Integrin αvβ3 is required for FGF1 to amplify TGF-β1-induced EMT. *A*, MCF10A cells were transfected with siRNA designed to target integrin β3 (si β3#1 and si β3#2). Non-targeting scramble siRNA (scr) is also transfected as a control. 24h post-transfection, cells were treated with 5 ng/ml TGF-β1 in the absence or presence of FGF1 (50 ng/ml) for 48h. Cells were then lysed, and protein levels were analyzed by Western blotting and probed with the indicated antibodies. *B*, MCF10A cells were transfected and treated same as above, confluent cells were then scratched by pipet tip, which allowed cells to migrate to the open space and cultured for additional 24h. Graph shows the fold change of closed wound area after 24 h. *C*, SK-BR-3 breast cancer cells stably transfected with shRNA for either integrin β3 (sh β3#5 and sh β3#12) or non-targeting scramble shRNA (scr), then cells were stimulated with 50 ng/ml FGF1 for 24h. Protein levels were determined by Western blotting with the indicated antibodies. *D*, SK-BR-3 cells were transfected and treated same as above, confluent cells were scratched and cultured for additional 24h. Graph shows the fold change of closed wound area after 24 h. *E*, ZR-75-30 breast cancer cells were stably transfected with integrin β3 expression vector or empty vector as a control, then cells were stimulated same as *C*. Protein levels were determined by Western blotting with the indicated antibodies. Data represent the mean ± S.E. (n = 3; *, *p* < 0.05). Bands intensity was measured by densitometry.

FGF1 directly interacts with integrin αvβ3 and induces the FGF1-FGF receptor (FGFR)-integrin αvβ3 ternary complex formation, which is necessary for FGF1 functions [20–22]. Gene silencing experiments of integrin β3 were performed to test the role of integrin αvβ3 on FGF1 and TGF-β1-inducing cell motility. Integrin β3 specific siRNAs effectively suppressed the gene expressions and suppressed TGF-β1-induced N-cadherin expression, but no change was observed in control siRNA (Fig 3A). The knockdown of integrin β3 decreased the effect of FGF1 on TGF-β1-induced cell motility to baseline (Fig 3B). These results suggest that αvβ3 is required for mediating FGF1 function in TGF-β1-induced EMT.

To confirm the importance of integrin αvβ3 to mediate FGF1 signaling in cancer cells, we establish stably knockdown of integrin β3 into SK-BR-3-breast cancer cells, in which αvβ3 is moderately expressed. Consistent with transient knock down of integrin β3 in MCF10A, its stable knockdown in SK-BR-3 cells suppressed FGF1-induced N-cadherin expression and the cell motility (Fig 3C and 3D). We overexpressed αvβ3 in ZR-75-30 cells, which hardly express αvβ3. Overexpression of αvβ3 in ZR-75-30 cells increased FGF1-induced N-cadherin expression (Fig 3E).

These results suggest that αvβ3 is required for FGF1 function in TGF-β1-induced EMT.

### Direct Interaction between FGF1 and Integrin αvβ3 Is Necessary for the Effect of FGF1 on EMT

**Fig 4:**
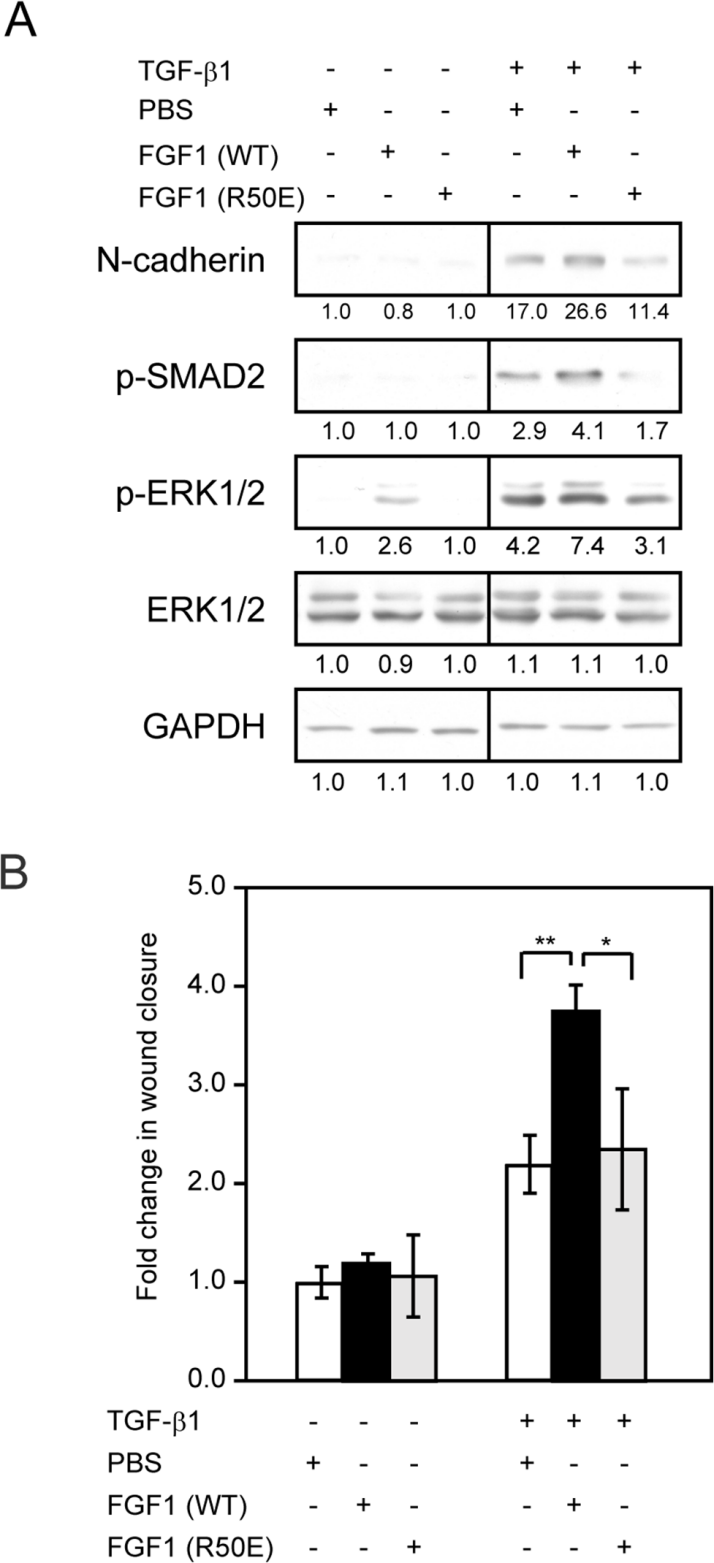
Integrin binding defective FGF1 mutant R50E is not able to enhance EMT. *A*, Starved MCF10A cells were treated with 5 ng/ml TGF-β1 in the presence of 50 ng/ml WT-FGF1 (WT) or integrin binding defective FGF1 mutant (R50E) for 48 h. Cell lysates were processed for Western blotting with the indicated antibodies. ERK1/2 and GAPDH served as a loading control. *B*, Starved MCF10A cells were treated with 5 ng/ml TGF-β1 in the presence of 50 ng/ml WT-FGF1 (WT) or integrin binding defective FGF1 mutant (R50E) for 24 h. Confluent cell monolayer was scratched by pipet tip and cultured additional 24 h in same conditions. Images were taken by phase-contrast microscopy and measured area of wound closure. Data represent the mean ± S.E. (n = 3; *, *p* < 0.05, **, *p* < 0.01). Bands intensity was measured by densitometry.

We have reported that an integrin-binding defective FGF1 mutant (Arg-50 to Glu, R50E) is defective in signaling functions [20]. We evaluated the role of direct binding of FGF1 to integrin αvβ3 in TGF-β1-induced EMT using R50E. MCF10A cells were treated with TGF-β1 in the absence or presence of WT-FGF1 or R50E. R50E did not increase N-cadherin, phosphorylated Smad2 and phosphorylated ERK1/2 levels induced by 5 ng/ml TGF-β1 (Fig 4A). Interestingly, FGF1 enhanced TGF-β1-mediated Smad2 phosphorylation. FGF1 alone does not induce Smad2 phosphorylation. If integrin αvβ3 is induced by TGF-β, FGF1 signaling is coupled to αvβ3 signals and may increase Smad2 signaling.

Wound healing assay also demonstrated that R50E did not show any effect on wound healing increased by TGF-β1 (Fig 4B). These findings suggest that the direct biding of FGF1 to integrin αvβ3 is crucial to the function of FGF1 to enhance TGF-β1-induced EMT.

### TGF-β1-Induced Loss of Polarity in 3D Culture of MCF10A Is Regulated by the Interaction between FGF1 and Integrin αvβ3

**Fig 5:**
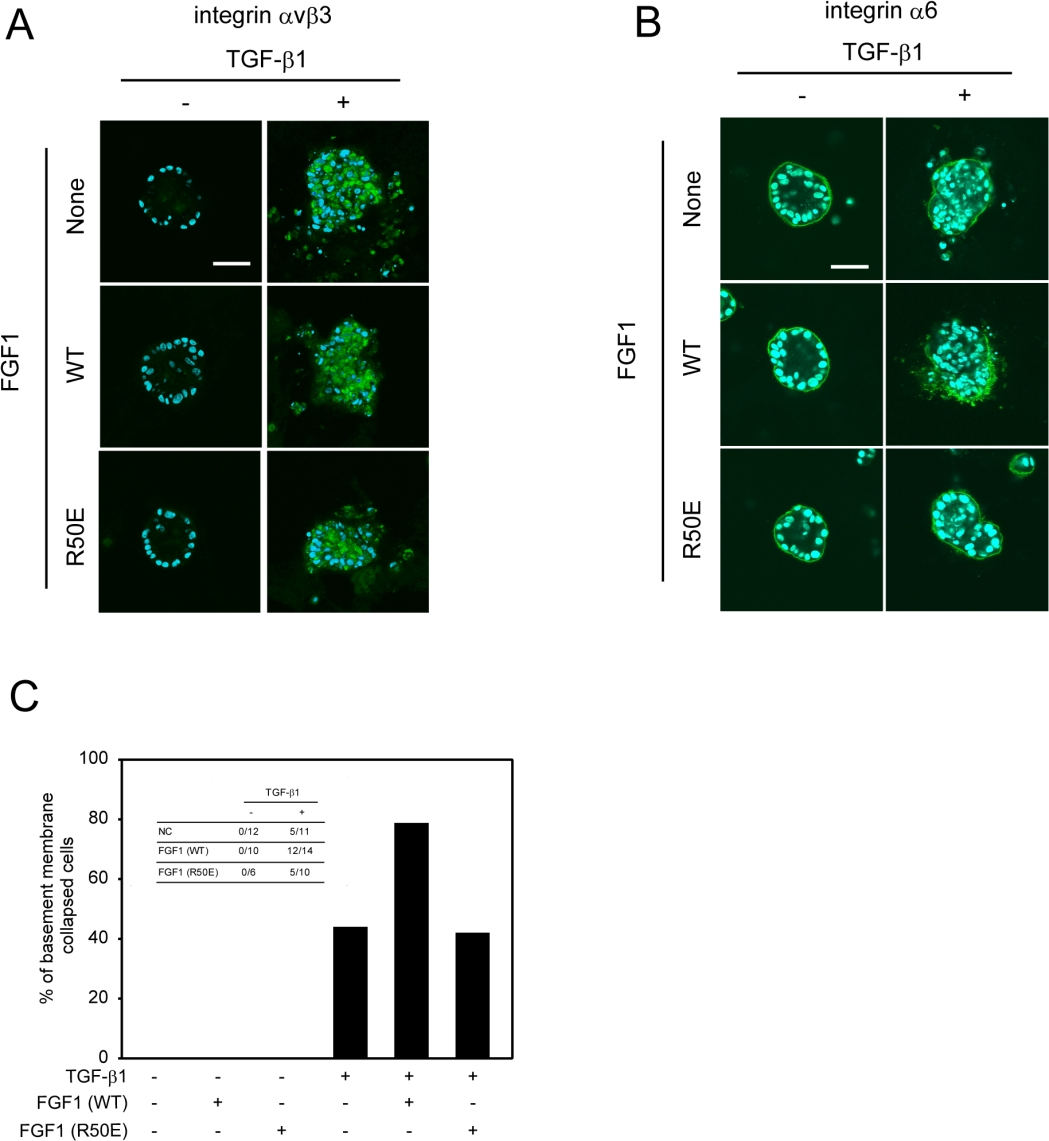
FGF1 augments TGF-β1-induced loss of polarity depends on the interaction between FGF1 and integrin αvβ3. *A and B*, MCF10A cells were seeded on glass cover slip coated with growth factor-reduced Matrigel. After 2 weeks of incubation, acinar structures were formed on the Matrigel. Assay medium was replaces to DMEM/F12 containing 2% horse serum and 2% growth factor-reduced Matrigel. Then cells were treated for inducting EMT with 5 ng/ml TGF-β1 together with either 50 ng/ml WT-FGF1 (WT) or integrin binding defective FGF1 mutant (R50E) for 4 days. The acini were immunostained with an anti-integrin αvβ3 (*A*, green) or an anti-integrin α6 (B, green) antibody together with DAPI labels nuclei (blue). Confocal images were acquired at the center plane of the acinar structure. Scale bar = 50 μm. *C*, MCF10A cells were treated as in *A*, then acini indicating discontinuous basement membrane was counted from the images.

Several oncogenes and excessive growth factors lead to disrupted mammary cell polarity in 3D sphere models [36]. We tested the functional roles of interaction between FGF1 and integrin αvβ3 in epithelial polarity using 3D culture of MCF10A cells. MCF10A cells were cultured on Matrigel to form acinus structure for 10 days, and then TGF-β1, FGF1, or both are added to the culture for additional 10 days. Immunofluorescence staining showed that integrin αvβ3 was not detected in non-treated MCF10A cells that form morphologically normal mammary acini with central lumen. When the cells were treated with 5 ng/ml of TGF-β1, integrin αvβ3 expression was induced mainly in cytosol, and the polarized organization was disturbed with cell-filled lumen (Fig 5A upper panels). FGF1 increased TGF-β1-induced depolarization of acinus structure at TGF-β1 (Fig 5A middle panels). However, R50E did not alter TGF-β1-induced depolarization (Fig 5A lower panels). Apicobasal polarization was detected by immunostaining for the α6 integrin as a basal marker. As observed above, R50E did not alter TGF-β1-induced depolarization (Fig 5B). We counted acini with a discontinuous basement membrane, namely collapsing polarized structure, based on integrin α6 expression against multiple acini in each condition (Fig 5C). We conclude that FGF1 enhances TGF-β-induced depolarization of acini structure. Thus, direct interaction between FGF1 and integrin αvβ3 is required to exert amplification effects of FGF1 on TGF-β1-induced EMT in MCF10A acinar structure.

### ERK Activation Is Required for Enhancement of TGF-β1-Induced EMT by FGF1

**Fig 6:**
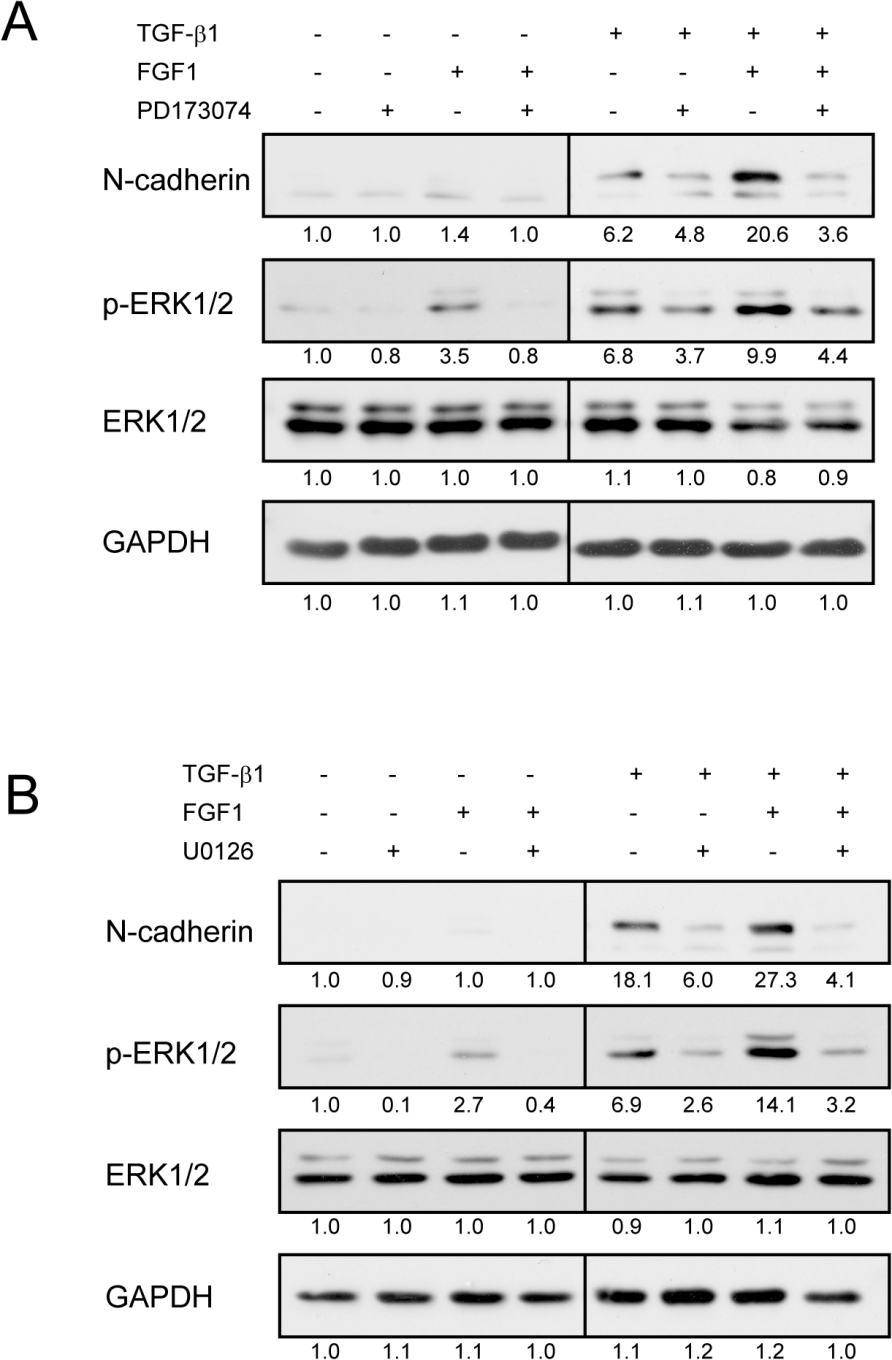
FGFR and ERK signaling is required for FGF1 to mediate TGF-β1 induced EMT in MCF10A cells. *A and B*, Starved MCF10A cells were stimulated with 5 ng/ml TGF-β1 and 50 ng/ml FGF1 in the presence of 1 μM PD173074 specific inhibitor of FGFR1 (A) or 10 μM U0126 specific inhibitor of MEK1 (B) for 48 h. DMSO was used as a solvent control for the chemicals. Cells were then lysed, and protein levels were analyzed by Western blotting with the indicated antibodies. Bands intensity was measured by densitometry.

We found that FGFR1 is up regulated by TGF-β but not FGFR2. These findings are consistent with the previous report [37]. PD173074, a selective inhibitor for FGFR, suppressed the effect of FGF1 on N-cadherin expression and ERK1/2 activation increased by TGF-β1 (Fig 6A, lane 8), suggesting that FGFR1 is necessary for enhancing effect of FGF1 on the N-cadherin level and ERK activation.

It is well known that FGF1 and TGF-β1 induce activation of the MAPK-signaling pathway in many cell types [33, 34]. To test if the MAPK pathway is involved in the effect of FGF1 on the TGF-β1-induced EMT, we treated MCF10A cells with U0126, a selective inhibitor for MAPK extracellular signaling-regulated kinase (ERK) kinase (MEK). The effect of FGF1 on N-cadherin expression and activation of ERK were significantly inhibited by U0126 (Fig 6B, lanes 8), indicating that the MAPK pathway is critical to transduce synergistic effect of FGF1 and TGF-β1 on EMT. Interestingly, TGF-β1-induced N-cadherin expression was suppressed by U0126 (Fig 6B, lanes 6), suggesting that TGF-β1-induced N-cadherin expression may depend mainly on MAPK pathway in MCF10A cells.

## Discussion

It has long been believed that integrins bind to extracellular matrix proteins, and growth factor receptors bind to growth factors, and the two separate signals are linked to one another in multiple steps in the pathway [17, 38, 39]. In contrast to this model, we reported that FGF1 directly binds to integrin αvβ3 and induces the FGFR1-FGF1-integrin αvβ3 ternary complex, and the interaction increases fibroblast and endothelial cell growth and motility [20, 22].

The present study demonstrated that TGF-β1 markedly induced integrin αvβ3 expression, and blocking integrin αvβ3 by RNAi abrogated FGF1- and TGF-β1-induced wound healing in MCF10A cells as well as in a breast cancer cell line that naturally expresses moderate level of integrin αvβ3. Thus, our findings suggest that integrin αvβ3 expression induced by TGF-β1 is required for enhanced EMT in mammary epithelial cells. Particularly, we showed that direct integrin interaction with FGF1 is involved in the increasing effect of FGF-1 on TGF-β1 signaling. Also, TGF-β1 induced FGFR1 expression, consistent with a report [37]. A specific inhibitor for FGFRs suppressed the enhancing effect of FGF1 on ERK activation and N-cadherin expression. These observations suggest that expression of αvβ3 and FGFR1 is required for the effect of FGF1 on TGF-β1-induced EMT.

Consistently, the integrin binding defective FGF1 mutant, R50E, did not augment TGF-β1-induced morphological change, N-cadherin expression and cell motility in MCF10A cells. Thus, the direct binding FGF1 to integrin αvβ3 is important for upregulation of EMT. Thus αvβ3 acts as a co-receptor of FGF1 and that FGF1 induces αvβ3-FGF1-FGF1 ternary complex formation [20, 21] in TGF-β1-induced EMT. If αvβ3 expression is not sufficient for FGF1 signaling, it is expected that FGF1 signaling is deficient. In our preliminary studies, αvβ3 expression was not enhanced when TGF-β1 was used at low concentrations (0.5 ng/ml) in MCF-10A cells. We observed that exogenous FGF1 did not enhance EMT, but rather suppressed it (data not shown). This underscores the important role of αvβ3 in FGF signaling. Also, R50E appears to antagonize TGF-β signaling. It is possible that FGF1 is required for TGF-β signaling, and R50E may inhibit it. Since FGF1 (and R50E) bind to all of FGF receptor family members and R50E may inhibit possible contribution of other FGF family members to TGF- β signaling. Further studies are needed to address this hypothesis.

The MAP kinase cascade is major signaling pathway involved in FGFR and integrin mediated cell fate. We found that the combination of FGF and TGF-β1 treatment resulted in more robust ERK activation compared to TGF-β1 alone or FGF1 alone. The MEK inhibitor blocked the activation of ERK signaling and upregulation of N-cadherin protein level caused by TGF-β1 and FGF1 combination. Similarly to the observation, TGF-β1 and EGF-induced EMT requires MAP kinase pathway, but not PI3K, p38, JNK or AP-1 pathway [40]. Our results show that stimulation with TGF-β only induced ERK activity. Several reports suggest the possibilities that TGF-β1 may induce autocrine FGF signaling [41], which can be inhibited by the FGFR inhibitor, and directly activate downstream of FGFR [33]. This may explain why the effects of specific inhibitor for FGFR were partial. Further studies of the crosstalk between TGF-β and FGF signaling by other methods will be needed to address these observations.

Several groups report that growth factors such as EGF, PDGF, HB-EGF, and insulin-like growth factor 1 (IGF1) increase TGF-β1-induced EMT in different cell lines [40, 42, 43]. These support our finding that synergistic effect of FGF1 and TGF-β1 on EMT. We recently reported that IGF1 signaling requires direct IGF1 binding to integrins such as αvβ3 [44–47] as in the case of FGF1. We propose that increased αvβ3 expression induced by TGF-β1 also plays a role in the enhancing effect of IGF1 on TGF-β1-induced EMT. On the other hand, vascular endothelial growth factor (VEGF), hepatocyte growth factor (HGF) and FGF suppress TGF-β1-induced EMT in kidney cortex cells and alveolar epithelial-like cells [13, 48–50]. Thus, it is unclear how/why different growth factors induce opposite effects on TGF-β-induced EMT. In the case of FGF, the levels of αvβ3 (and perhaps other integrins) might determine whether or not FGF augments TGF-β-induced EMT, as described above. It would be interesting to study if the levels of integrin expression and integrin expression profiles may be critical factors in the effect of other growth factors on TGF-β-induced EMT in different cell lines in future studies.

In conclusion, the present study provides a new insight into the role of integrin αvβ3 in the enhancing effect of FGF1 in TGF-β1-induced EMT. We provides evidence that direct FGF1 binding to integrin αvβ3 is critical for the enhancing effect of TGF-β1-induced EMT by FGF in mammary epithelial cells.
